# A snap shot of the short-term response of crustaceans to macrophyte detritus in the deep Oslofjord

**DOI:** 10.1038/srep23800

**Published:** 2016-03-30

**Authors:** Eva Ramirez-Llodra, Eli Rinde, Hege Gundersen, Hartvig Christie, Camilla With Fagerli, Stein Fredriksen, Janne Kim Gitmark, Karl Norling, Mats Gunnar Walday, Kjell Magnus Norderhaug

**Affiliations:** 1 Norwegian Institute for Water Research (NIVA), Gaustadalléen 21, NO-0349 Oslo, Norway; 2 Department of Biosciences, University of Oslo, PO Box 1066 Blindern, NO-0316 Oslo, Norway; 3 Department of Biological and Environmental Sciences, University of Gothenburg, SE-41319 Gothenburg, Sweden

## Abstract

A test deployment of a time-lapse camera lander in the deep Oslofjord (431 m) was used to obtain initial information on the response of benthic fauna to macroalgal debris. Three macroalgal species were used on the lander baited plate: *Fucus serratus, Saccharina latissima* and *Laminaria hyperborea* and observed during 41.5 hours. The deep-water shrimp *Pandalus borealis* were attracted to the macroalgae rapidly (3 min after the lander reached the seafloor), followed by amphipods. Shrimp abundances were significantly higher in areas covered by macroalgae compared to the adjacent seafloor and the number of shrimp visiting the macroalgae increased with time. Amphipods arrived 13 hours later and were observed mainly on decaying *L. hyperborea*. The abundance of amphipods on *L. hyperborea* increased rapidly, reaching a peak at 31 h after deployment. These initial observations suggest that debris from kelp forests and other macroalgal beds may play an important role in fuelling deep benthic communities in the outer Oslofjord and, potentially, enhance secondary production of commercial species such as *P. borealis.*

The maintenance and functioning of most deep-sea ecosystems depend on organic matter produced in the euphotic zone. Often, studies on input of such organic matter to the deep seafloor have focused on material derived from phytoplankton blooms and zooplankton grazers and predators (phytodetritus, faecal pellets) that slowly sinks and reaches the seafloor as a particulate rain^1^. Additionally, there is an increasing awareness of the importance of benthic primary production in macroalgal beds in the euphotic zone and their role in supporting ecosystems both in shallow and deep waters, including deep fjords^2^,^3^. Macroalgae act as foundation species in costal zones of temperate and polar waters and support high secondary productivity of rich and diverse communities of invertebrates, fish and marine mammals, including many commercially-important species^4^,^5^. Along the Norwegian coast, the kelp habitat covers 18000 km^2^ with a calculated biomass of 180 million tonnes^6^. Kelp laminas are shed every year and this biomass is approximately equivalent to the annual kelp production. Consequently, kelp plants produce large amounts of particulate organic carbon (POC) and dissolved organic carbon (DOC) through the exudation and steady erosion of distal parts of the lamina or loss of whole plants during storms^7^,^8^. Between 80–90% of kelp production is estimated to be lost from the kelp forests and exported to shallow- and deep-water ecosystems^9^. However, according to a recent review by Krumhansl and Scheibling^2^, a comprehensive understanding of the nature and extent of this subsidy is still lacking. A recent study in an Arctic fjord indicates that macroalgal detritus can represent an important contribution to benthic food webs, challenging well-accepted paradigms of pelagic-benthic coupling in Arctic shelf ecosystems and highlighting the need for thorough analyses of carbon baselines in food-web studies^3^.

In this study, we used the opportunity provided by a single test deployment of a time-lapse camera lander in the deep Oslofjord, to obtain a snapshot of the response of the local benthic megafauna to experimentally introduced macrophyte detritus.

## Material and Methods

### Study area

**Figure 1:**
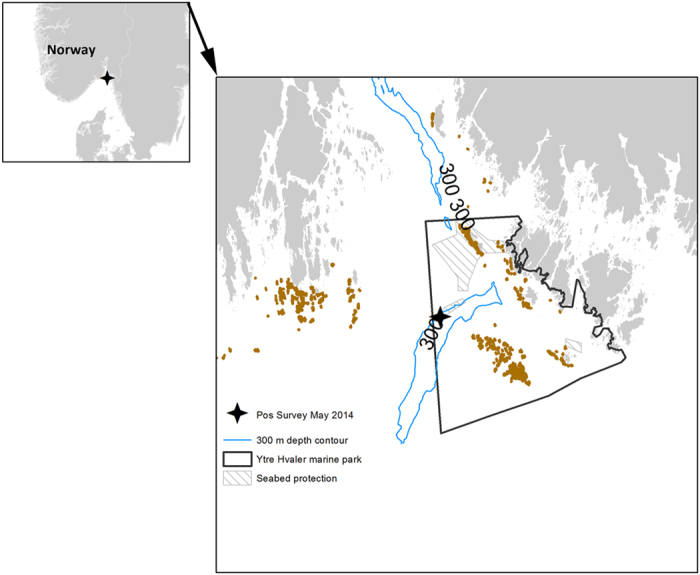
Map showing the general location of the Outer Oslofjord with the study area (black star) and the details of the study area. Black line delimits the Ytre Hvaler National Park; Stripped polygons delimit the areas with full protection (i.e. no fishing or other leisure or commercial activities); Brown areas indicate areas with kelp forest. The 300 m isobaths is shown. The figure was made using the software ArcMap 10.1 by ESRI (http://www.esri.com/).

A test-deployment of a time-lapse camera lander was conducted on the deep (431 m) outer Oslofjord from R/V Trygve Braarud on May 2014, within a protected seabed area of the Ytre Hvaler National Park (Fig. 1). This is a deep, relatively flat, soft-sediment area that has full protection from trawl fisheries. It is located downstream from existing kelp forests and is situated close to an important fishery area for the shrimp *Pandalus borealis.*

### Time-lapse camera deployment

**Figure 2:**
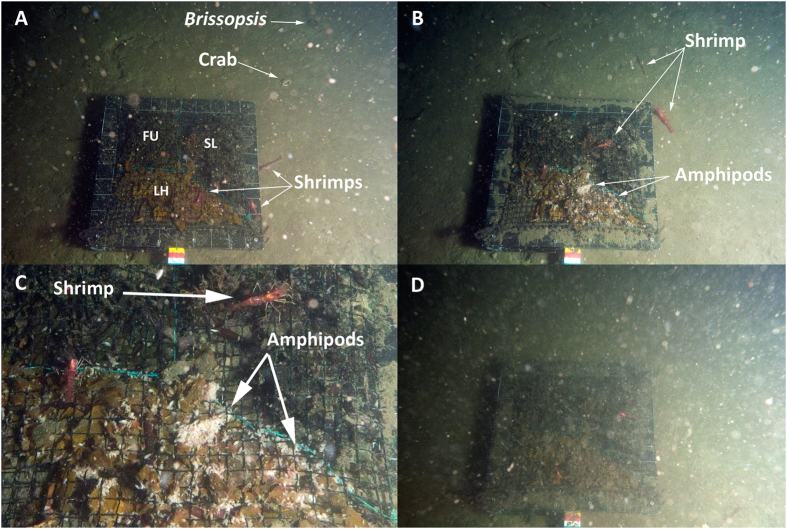
Snap shots taken with the TLC showing the bait plate with the 3 algal treatments and the adjacent sediment. (**A**) The TLC bait plate on the seafloor at t = 300 min showing the three algal areas and benthic fauna. FU, *Fucus serratus*; SL, *Sacharina latissima*; LH, *Laminaria hyperborea*. (**B**) The TLC bait plate on the seafloor at t = 1515 min showing shrimp and amphipods. (**C**) Detail of the bait plate at t = 1515 min showing a close up of one shrimp and the amphipods on the *L. hyperborea* treatment. (**D**) The TLC bait plate on the seafloor at t = 915 min showing the re-suspension event.

The Time-Lapse Camera (TLC) used is a free-fall lander with a DCS12000 Ocean Imaging Systems camera housing, control and strobe with a Nikon D90 SLR camera mounted on a tripod, with a bait plate in front of the camera at seafloor level. The bait plate was divided into three areas (Fig. 2A), one for each of three macroalgal species. Area FU (0.06 m^2^) contained fresh brown algae (*Fucus serratus*) collected from Solbergstrand in the inner Oslofjord and kept in a bucket with seawater for 1 day. Area SL (0.08 m^2^) contained *Saccharina latissima* that had been collected in the middle part of the Oslofjord (Jeløya Island) and kept in running seawater for 16 days. Area LH (0.1 m^2^) contained *Laminaria hyperborea* collected from the middle part of the Oslofjord (Drøbak) and kept in a closed bucket with seawater for 7 days. As this was a test deployment, available algae at the time of the cruise were used. Consequently, no systematic degradation of the macrophytes was followed and thus no quantitative measure of degradation was available. Qualitatively, *L. hyperborea* was the most degraded alga, followed by *S. latissima* and *F. serratus*.

The total time of the TLC on the seafloor was 41.5 hours. The camera settings used were: ISO = 640, SS = 1/60, F.8. One photo was taken every 3 minutes resulting in a total of 825 photos. Of these photos, 805 were taken on the seafloor and 20 photos were taken during descent and ascent, which were not included in the analyses. For the analyses, t = 0 represents the first photo taken on the seafloor.

### Photo processing

All photographs were analysed using Adobe Photoshop CS5. All observed megafauna were identified to the lowest possible taxonomic level. The dominant fauna (shrimp and amphipods) were counted in each of the three algal areas (FU, SL and LH) on the bait plate as well as in three areas of the same size and shape as the algal areas in the adjacent seafloor, where no macroalgal debris was observed on the sediment. The shrimp were counted in each photo, whereas, due to high abundance of amphipods, these were quantified in each photo during the first 16 h of deployment, every 10 photos until t = 25 h and every 20 photos from t = 25 h to the end. The shrimp and amphipod counts in each photo were standardised per area (m^2^) to provide density values (individuals.m^−2^).

### Generalised additive mixed effects models (GAMMs)

GAMs can be used to model trends as a smooth, nonlinear function of time, and they provide a framework for testing the statistical significance of changes in abundance^10^. We performed Generalized Additive Mixed Model (GAMM) analyses of shrimp and amphipod density (family: poisson) as a function of the duration of the deployment (R version 3.1.1 and the *mgcv* library,^11^). Treatment (four levels: the three algal species on the bait plate with 1 replicate, and the adjacent no-algae/no-lander area with 3 replicates) was included as a fixed factor in the shrimp analysis. The three no-algae/no-lander areas allow testing the effect of ‘lander with algae’, while adjusting for the temporal autocorrelation among the counts within the bait plate on the lander and within the no-algae area. In the amphipod analysis, only the counts on the bait plate were used, since no amphipods were observed on the adjacent sediment. Temporal autocorrelation between the observations in the bait plate and in the no-algae/no-lander areas on the adjacent sediment was tested and controlled for by including a one-lag temporal auto**-**regressive error structure grouped for each area. Visual inspection of residual plots did not reveal any obvious deviations from homoscedasticity or normality.

## Results

### General observations

The fauna observed in the photographs included mostly shrimp, *Pandalus borealis*, and un-identified amphipods (Fig. 2A–C). Additionally, three fish, eight ctenophores, two medusae and a salp were observed swimming, while two crabs and two irregular echinoids of the genus *Brissopsis* were observed on the adjacent seafloor (Fig. 2B). Three minutes after the TLC reached the seafloor, the first shrimp was observed on the bait plate, in the *Fucus serratus* area (FU). Of the 805 photos analysed, shrimp were observed on the algal areas (FU, SL and/or LH) in 94.3% of the photos, whereas 39.4% of the photos showed shrimp on the adjacent sediment (see Supplementary Fig. S1). Amphipods arrived on the bait plate at t = 801 min (13h21′) and were present on the bait plate at all times afterwards, with a clear preference for *Laminaria hyperborea* (Fig. 2B,C and Supplementary Fig. S2). No amphipods were observed on the adjacent sediment areas at any time. At t = 900 min (15 h, between 07:44 and 08:14 AM on day two), a re-suspension event was observed, lasting for 30 min, where the bait plate was almost not visible (Fig. 2D).

### Abundance variation with time

**Figure 3:**
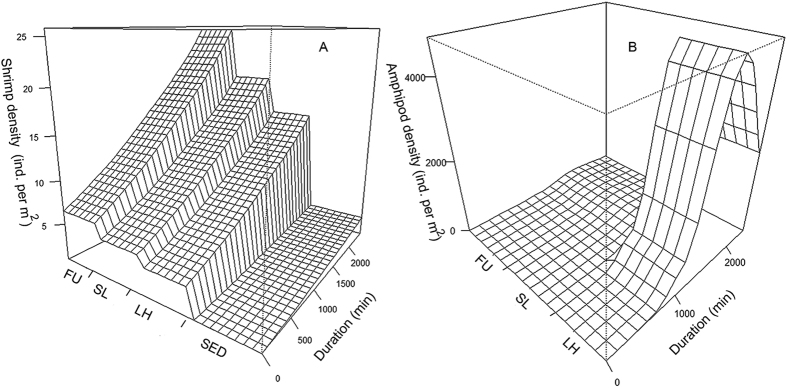
GAMM models showing changes in density of shrimp (**A**) and amphipods (**B**) as a function of duration of study and algal treatment. FU: *Fucus serratus*; SL: *Saccharina latissima*; LH: *Laminaria hyperborea*; SED: sediment (no algal/no lander treatment).

Temporal autocorrelation was found (significantly reduced AIC values, Burnham *et al.*, 2011) and accounted for in both the shrimp and the amphipod GAM. The shrimp model showed a steady increase in shrimp density with time on each of the algal species, but not on the adjacent sediment (R_adj_ = 0.34, Fig. 3A). The amphipods showed a delayed and marked response with time on the alga *Laminaria hyperborea* and only small differences on the other algal species (R_adj_ = 0.96, Fig. 3B). The steep increase in amphipod density reaches a maximum after about 1500 min (25 hours), before the density is reduced towards the end of the TLC deployment.

## Discussion

The results presented here provide the first *in situ* observations indicating that macroalgal debris may fuel deep-water communities in the Oslofjord. The supply of macroalgal and wood debris to deep seafloor has been known since the Challenger Expedition in the late 19^th^ Century^12^. Such supplies increase local heterogeneity by creating patches of enriched sediment that modify the structure of the local benthic community and increase α and β-diversity^1^,^13^. Several studies have shown that organic debris, including macroalgae, can accumulate at high rates in habitats such as submarine canyons and fjord basins, driven by the specific hydrodynamics of these habitats^14^. This accumulation can affect the functioning of the local ecosystem, including food webs^3^. For example, increased megafauna diversity and density and higher fish abundance have been reported from submarine canyons in California and Hawaii and these patterns have been related to higher food availability in the canyons, including macrophyte detritus^14^,^15^. Harrold *et al.*^16^ suggested that the input of kelp-derived organic matter in a submarine canyon off California has a direct impact in the reproductive success of the dominant deep-sea echinoid *Allocentrotus fragilis*. The observations in the Oslofjord showed a rapid and contrasting response in shrimp and amphipod abundance to the supplied macroalgal debris. The shrimp, *Pandalus borealis*, responded immediately and increased in abundance on each of the three algae species throughout the time of deployment. In contrast the amphipods occurred later and were mainly attracted to the most degraded macroalgal species, *Laminaria hyperborea*. This differential response caused a clear, visual border on the bait plate between the *L. hyperborea* area, covered by high densities of amphipods, and the *S. latissima* area, with low amphipod density (Fig. 2C). The preference for *L. hyperborea*, which was supplied at the most degraded level, could reflect a trophic relationship with the algae as bacterial degradation of kelp may increase the food value and accessibility to consumers^17^.

A scavenging experiment in Sognefjord (west Norway) using jellyfish and fish as bait has demonstrated the rapid response of Atlantic hagfish, galatheid crabs, amphipods and shrimp, fully consuming the jellyfish carcasses within 2.5 hours^18^. In another *in situ* study using phytodetritus pulses^19^, Jeffreys *et al.* demonstrated that deep-sea fish consume experimentally deployed phytodetritus in the NE Atlantic at 3000 m depth, while in the deep Mediterranean, it was the invertebrate fauna that consumed, at a slower rate, the same food supply. In our study, no visual evidence of macrophyte consumption from the shrimp or the amphipods was observed. This is not surprising, however, as the macroalgae provided were not highly degraded and, thus, more difficult to exploit as a food source. Additionally, the lack of replication and control treatment in our study limits our capacity to assess a potential “artificial reef” effect of the lander in the ecosystem^20^, as well as the effect of algal species or degradation stage. However, the study demonstrates a difference in response between shrimp and amphipods, both in the time of arrival and over time, as well as a differential influence of the algae on the amphipod abundance response, suggesting a trophic relationship.

Additionally to the biological observations, we witnessed a strong 30 minute re-suspension event, with unknown origin. The study area is within a protected seafloor zone of the Ytre Hvaler National Park, where shrimp trawling is not permitted. However, the nearby area is an important fishing ground for the shrimp *Pandalus borealis.* Hence, a possible cause could be the presence of a trawler operating in this region. In the NW Mediterranean, it has been demonstrated that re-current daily sediment gravity flows are triggered by commercial trawling, resulting in the deposition of large quantities of sediment several kilometres downslope^21^,^22^. Another alternative could be an adjacent sediment slide.

Our results indicate that regular input of kelp detritus to the deep Oslofjord could have a significant influence in fuelling the benthic megafauna, including shrimps of commercial value. However, further studies are necessary to quantify the potential macroalgal consumption by the benthic fauna, and a robust experimental design is needed to isolate the effects of algal species, degradation state and of the lander itself. Describing the role played by macroalgae in fuelling deep-sea communities is important in understanding the potential deep-sea ecosystem response to kelp regime shifts induced by climate change or other stressors^23^.

## Additional Information

**How to cite this article**: Ramirez-Llodra, E. *et al.* A snap shot of the short-term response of crustaceans to macrophyte detritus in the deep Oslofjord. *Sci. Rep.* **6**, 23800; doi: 10.1038/srep23800 (2016).

## Supplementary Material

Supplementary Information
