# Supplementary material for: A snap shot of the short-term response of crustaceans to macrophyte detritus in the deep Oslofjord
Source: Sci Rep. 2016 Mar 30;6:23800. doi: 10.1038/srep23800 (PMC4824494; doi:10.1038/srep23800)

A snap shot of the short-term response of crustaceans to macrophyte detritus in the deep Oslofjord

Eva Ramirez-Llodra[1,*], Eli Rinde[1], Hege Gundersen[1], Hartvig Christie[1], Camilla With Fagerli[1], Stein Fredriksen[2], Janne Kim Gitmark[1], Karl Norling[1,3], Mats Gunnar Walday[1], Kjell Magnus Norderhaug[1,2]

SUPPLEMNETARY MATERIAL

Supplementary Figure S1. Plot showing the change in shrimp abundance with time in the three algal treatments and the adjacent sediment. FU, *Fucus serratus*; SL, *Saccharina* latissima; LH, *Laminaria hyperborea*; SED, sediment.

Supplementary Figure S2. Plot showing the change in amphipod abundance with time in the three algal treatments. No data on the sediment are shown as no amphipods were observed on the sediment adjacent to the bait plate. FU, *Fucus serratus*; SL, *Saccharina* latissima; LH, *Laminaria hyperborea*.

Supplementary Figure S1

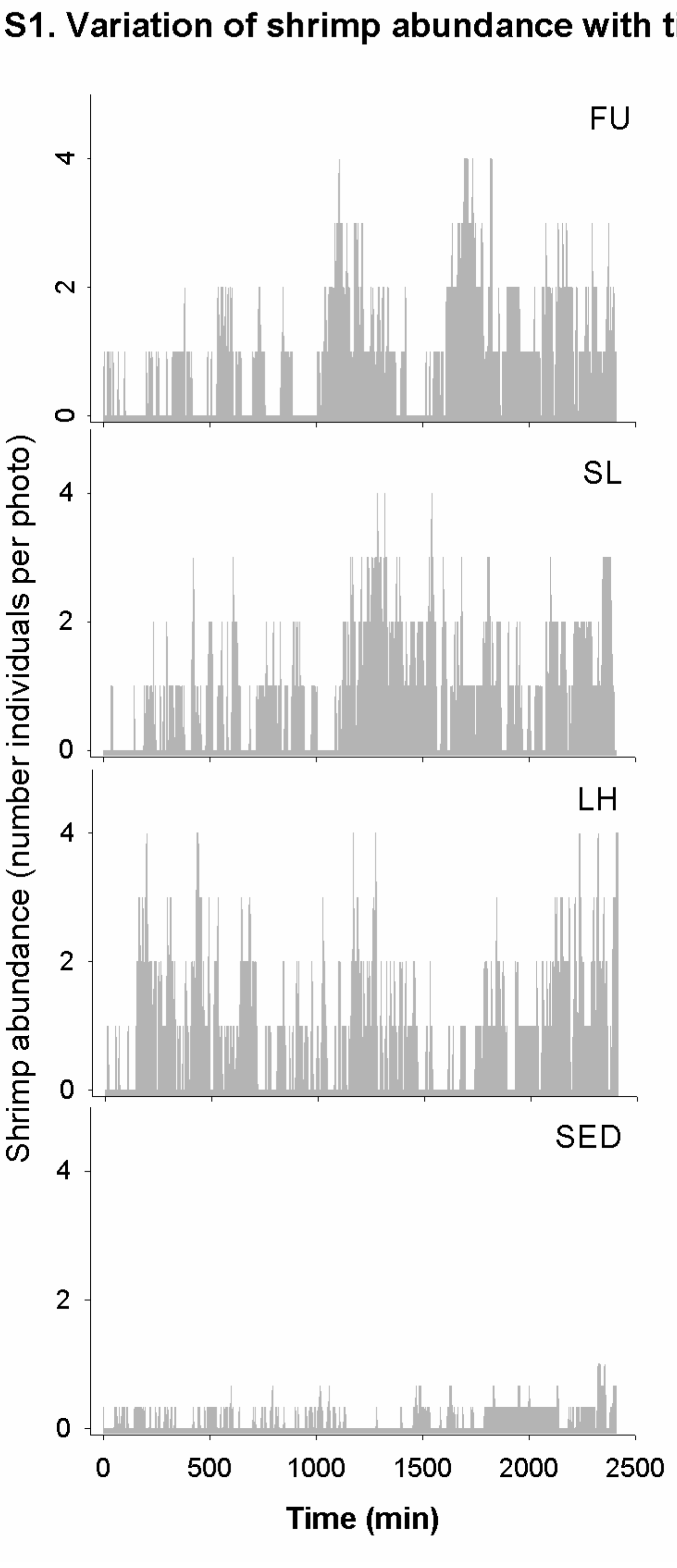

Supplementary Figure S2

S2. Variation of amphipod abundance with time

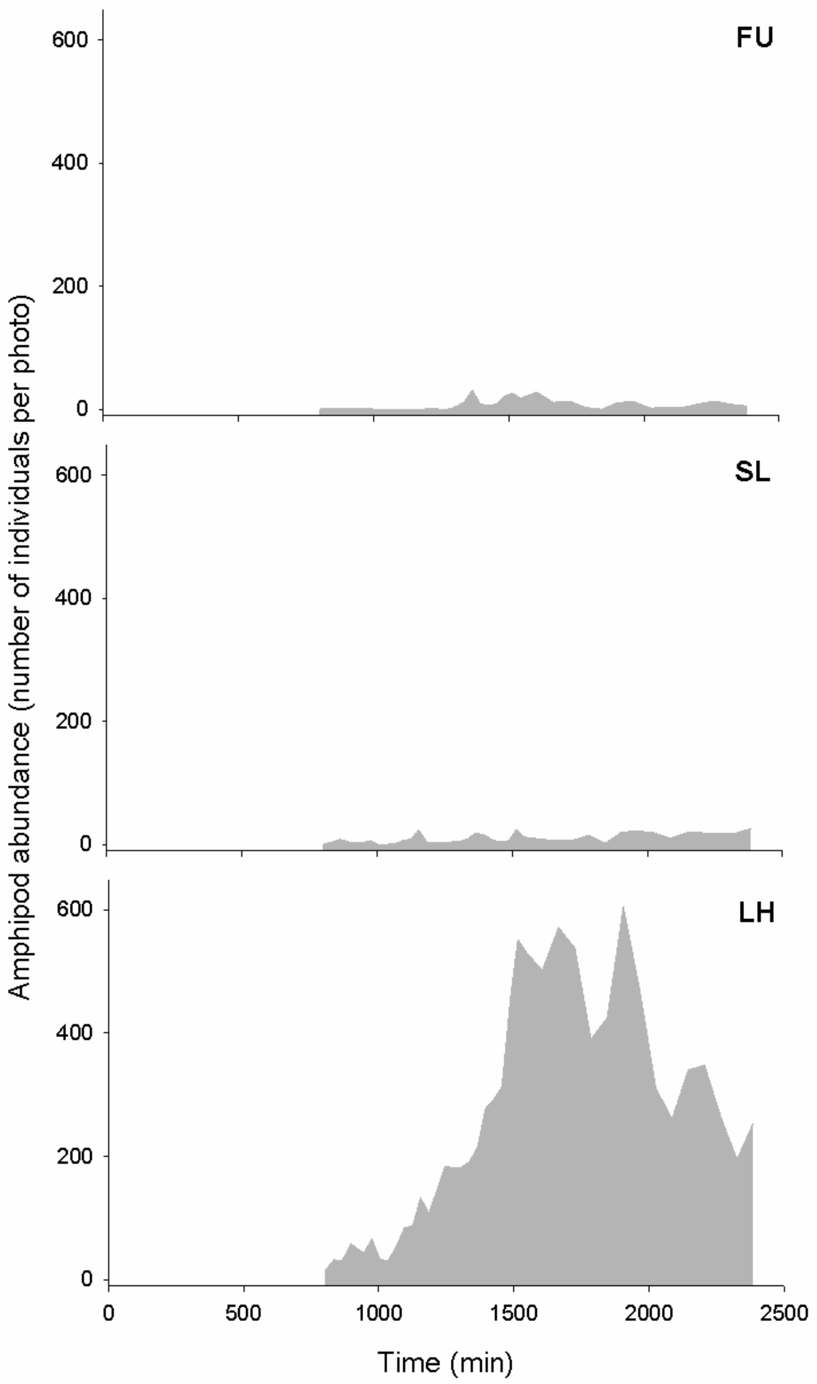

Supplement: Supplementary Information [file srep23800-s1.pdf]
